# Response Surface Methodology Based Optimization of Test Parameter in Glass Fiber Reinforced Polyamide 66 for Dry Sliding, Tribological Performance

**DOI:** 10.3390/ma15196520

**Published:** 2022-09-20

**Authors:** Narendran Jagadeesan, Anthoniraj Selvaraj, Santhosh Nagaraja, Mohamed Abbas, C. Ahamed Saleel, Abdul Aabid, Muneer Baig

**Affiliations:** 1 Mechanical Engineering Department, Paavai College of Engineering, Namakkal 637018, India; 2 Information Science and Engineering, MVJ College of Engineering, Bangalore 560067, India; 3 Department of Mechanical Engineering, MVJ College of Engineering, Near ITPB, Whitefield, Bangalore 560067, India; 4 Electrical Engineering Department, College of Engineering, King Khalid University, Abha 61421, Saudi Arabia; 5 Electronics and Communications Department, College of Engineering, Delta University for Science and Technology, Gamasa 35712, Egypt; 6 Department of Mechanical Engineering, College of Engineering, King Khalid University, Abha 61421, Saudi Arabia; 7 Department of Engineering Management, College of Engineering, Prince Sultan University, Riyadh 11586, Saudi Arabia

**Keywords:** polyamide, GFRPA66, composite, SWR, COF, ANOVA

## Abstract

The tribological performance of a glass fiber reinforced polyamide66 (GFRPA66) composite with varying fiber weight percentage (wt.%) [30 wt.% and 35 wt.%] is investigated in this study using a pin-on-disc tribometer. GFRPA66 composite specimens in the form of pins with varying percentages of fiber viz., 30 wt.% and 35 wt.% are fabricated by an injection molding process. Tribological performances, such as coefficient of friction (COF) and the specific wear rate (SWR), are investigated. The factors affecting the wear of GFRPA66 composites [with 30 wt.% and 35 wt.% reinforcements] are identified based on the process parameters such as load, sliding velocity, and sliding distance. Design Expert 13.0 software is used for the experimental data analysis, based on the design of experiments planned in accordance with the central composite design (CCD) of the response surface methodology (RSM) technique. The significance of the obtained results are analyzed using analysis of variance (ANOVA) techniques. To attain minimum SWR and COF, the wear performance is optimized in dry sliding conditions. The analysis of experimental data revealed that SWR and COF increased with increasing load, sliding velocity, and sliding distance for GFRPA66 [30 wt.%], but decreased with increasing polyamide weight percentage. The SWR for a maximum load of 80 N, and for a sliding velocity of 0.22 m/s, and a sliding distance of 3500 m for GFRPA66 composite specimens with 30 wt.% reinforcements are found to be 0.0121 m^3^/Nm, while the SWR for the same set of parameters for GFRPA66 composite specimens with 35 wt.% reinforcements are found to be 0.0102 m^3^/Nm. The COF for the GFRPA66 composite specimens with 30 wt.% reinforcements for the above set of parameters **is** found to be 0.37, while the GFRPA66 composite specimens with 35 wt.% reinforcements showed significant improvement in wear performance with a reduction in COF to 0.25. Finally, using a scanning electron microscope (SEM), the worn surfaces of the GFRPA66 are examined and interpreted.

## 1. Introduction

Polymers are now widely used in the manufacture of various components such as machine parts, cams, bearing, bearing cages and gears, worm wheel parts used in gear box, etc., in various fields of engineering such as automobiles, robots, and aircrafts. They have significant advantages such as a low friction coefficient without external lubrication, improved abrasion, corrosion, and wear resistance, low density, and suitability for mass production [1]. Nowadays, many tribological applications are using polyamide and polyamide composites as a better alternative to metallic parts in the manufacturing of the mechanical parts. Weight, cost, and ease of manufacturing are also often considered as additional benefits for using polyamides in composites [2].

Polyamide has a highly ordered (semi-crystalline) molecular structure, a hydrogen bond, and superior wear and mechanical properties due to its semi-crystalline nature [3,4]. Understanding wear mechanism and behavior under different sliding conditions is critical when using polyamide and polyamide composites for sliding applications [5]. Moreover, the adhesion mechanism of friction of polymer occurs due to the breakage of weak bonds between polymer pin and steel disc. Similarly, dissipation of energy in the contact area of the deformation mechanism is a major challenge [6,7,8]. The nylon gears and bearings possess hydrogen bond and the Van der Waals force present in the molecular chains resist the wear [9,10]. The transfer layer protects wear loss of material, and this is controlled by adhesion and cohesion of the transfer film. The wear loss of disc depends on the transfer layer formation and surface degradation occurs due to the loss of transfer layer [11].

The need to withstand the environmental degradations and higher stress are also the major concerns for plastic parts in current scenario [12,13,14]. To overcome the above challenges, mechanical and tribological properties such as tensile strengths, Young’s modulus of polyamide and bending strength needs to be improved by using fillers such as glass fiber, MoS_2_ (Molybdenum disulfide), and carbon fiber in a polymer matrix [15,16,17,18]. On the other hand, while improving mechanical and tribological properties, several properties tend to decrease, owing to the improper distribution of reinforcements in the matrix phase. Hence, there is a need for filler material. The filler may be affected by fiber reinforcement. The filler material used depends on the matrix, size, shape and type of the reinforcement. The tribological characteristics of such composites mainly depends on wear conditions.

Polyamide 66 (PA66) is preferred due to its economy, maximum wear resistance, better strength, thermal properties, and capability to absorb energy and plastically deform without fracturing [19,20]. For ultimate properties, PA66 is used in food and chemical industry, at the same time it is used for betterment of tribological properties facilitating their use in components such as gears, bearings etc. [21,22,23,24], and also polyamide has inherent lubrication property as added advantage [25]. Many researchers have explained the effect of adding glass fiber as a reinforcement material to PA66 for enhancing its wear behavior The reinforcement material, such as glass fiber, increases wear resistance and generates a transfer film between the polyamide pin and steel disc during surface contact, as well as it acts as a self-lubricant [26].The wear behavior of polyamide 66 and polyamide composites with 10, 20, and 30 wt.% glass fiber addition have been studied. The results show an increase in wear resistance with an increase in weight percentage [27]. The ultimate strength, flexural strength, and elastic modulus all increase as the weight fraction of glass fiber increases [28]. As a result, the deformation mechanism of polymer should be considered while using fiber reinforced material. The deformation mechanism mainly depends on tribological properties. At low load and low sliding speeds, the coefficient of friction and wear becomes high, but as the load increases within the elastic limit, the friction coefficient decreases. With a further increase in the load beyond the elastic limit, the friction coefficient may increase due to the increase in the plastic deformation [29]. However, the tribological properties are found to be lower while using a glass fiber reinforced polymer composite with 30 weight % glass fiber and 6 wt.% nylon (30 wt.% GFR and 6 wt.% nylon) [30].

Also, several studies have focused on the optimization of the reinforcement percentage using statistical methods. The best statistical method such as design of experiments (DoE) for determining a specific quality characteristic of a result by using a large number of variables is an important technique for optimization studies. DoE consists of arrangement of variables, experiments, performance, evaluation, experiments controlled by a set of data facilitating the reduction in the number of experimental trials [31]. This supports the primary goal of the current study, which is to investigate the effect of process parameters and condition optimization using RSM for optimizing the tribological performance of polyamide. This fosters the objective of the optimization of process parameters such as load, temperature, weight percentage of reinforcement, sliding velocity, sliding distance, and etc., especially using the response surface methodology (RSM) [32]. To optimize the process variables for tribological performance, the central composite design (CCD) is used, and the results of the experiments are scientifically tabulated. The influence of process parameters of polyamide with varying weight percentages are identified and also the number of trials is reduced via RSM to improve the tribological performance [33,34,35,36,37]. Also, several related studies on the optimization of process variables using the RSM approach for improving the tribological performance in polyamide has been studied and adopted for the present work [38,39,40,41,42,43,44,45,46,47,48,49,50,51].

This study focuses on coefficient of friction (COF) and the specific wear rate (SWR) of PA66 composite with 30 wt.% and 35 wt.% glass fiber against a very high strength alloy steel (EN31) disc. The glass fiber reinforced polyamide 66 composite with 30 and 35 wt.% reinforcement has been preferred due to superior mechanical properties such as increased strength, rigidity, creep strength, and dimensional stability [52,53,54,55,56]. When compared to unreinforced PA66, the properties of the glass filled composites make it suitable for use in parts subjected to high static loads for extended periods of time in high temperature conditions [57,58,59,60,61]. The following assumptions are considered for investigation: (1) while increasing the weight percentage of glass fiber, the COF and SWR decreases; (2) if the load, sliding velocity and sliding distance is increased, the transfer film in the specimen is not affected by contact surface temperature; (3) if the weight of glass fiber increases it may increase elastic modulus and ultimate strength of glass fiber. Finally, a study was conducted to analyze the optimized conditions in order to determine the tribological performance of polyamide and also results are validated by RSM.

## 2. Materials and Methods

### 2.1. Materials

In this investigation, the effect of glass fiber reinforcement on the tribological properties of the PA66 are analyzed. Henceforth E Glass fibers (E glass) were obtained from Ikon traders, Bangalore, and used as reinforcements, while the PA66 matrix was sourced from SS Impex, Bangalore, and used for the synthesis of the composite. The Toshiba make injection molding machine was used to create the eighteen polyamide pins to determine the tribological properties.

### 2.2. Specimen Details

**Figure 1:**
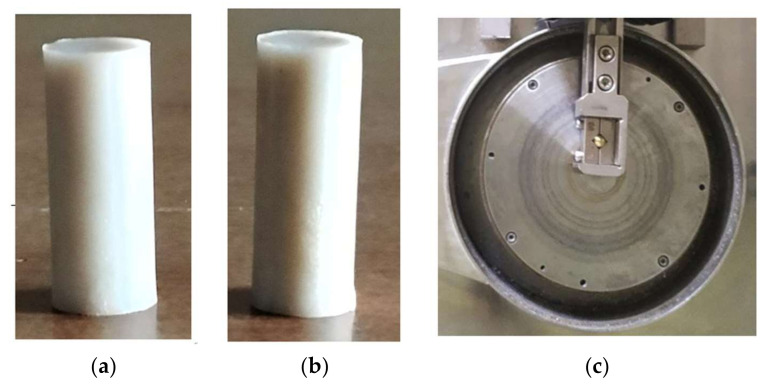
(**a**) GFRPA66 30 wt.% pin, (**b**) GFRPA66 35 wt.% pin, and (**c**) EN31 steel disc.

The GFRPA66 pin with varying weight percentage of reinforcements viz., 30 wt.% and 35 wt.% of E Glass fiber is as shown in Figure 1a,b. The dimensions of cylindrical polyamide pins were 12 mm × 30 mm and EN31 steel disc specimen was 165 mm in diameter and 8 mm in thickness and is depicted in Figure 1c.

## 3. Experimental Design

### 3.1. Friction and Wear Analysis

**Figure 2:**
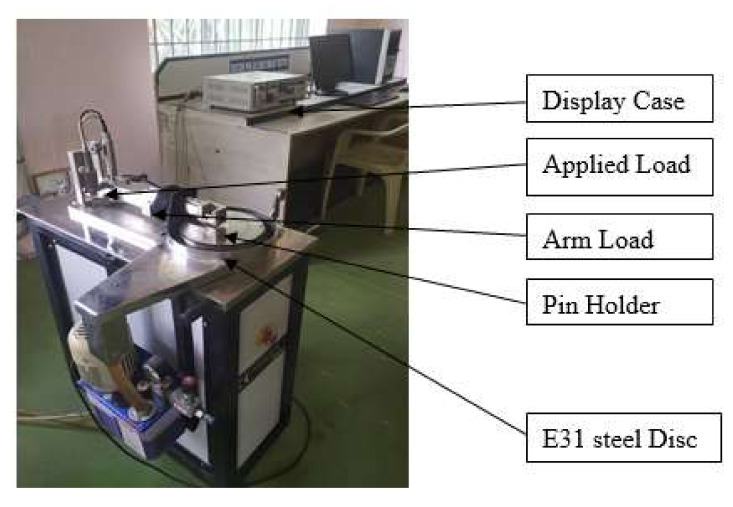
DUCOM Pin on Disc Tribometer.

The COF and SWR of GFRPA66 with 30 wt.% and 35 wt.% reinforcements are studied under dry conditions by using pin-on-disc tribometer (DUCOM make) which is as shown in Figure 2. The input parameters for tribological tests are load (60, 70, and 80 N), sliding velocity (0.16, 0.19, and 0.22 m/s) and sliding distance (2500, 3000, and 3500 m). The experiments are conducted with constant room temperature under dry sliding conditions. Before conducting the test, the disc was cleaned by acetone and surface of the pins were polished using emery paper (320 and 600 grit sizes) [48]. Subsequently, the friction force was measured, and the data were recorded during the experiment by using transducer. The mass losses of the pin were measured by using micro weighing balance with an accuracy of 0.0001 mg. The mass loss was determined by measuring the mass of the specimen before and after the experiment.

### 3.2. Response Surface Methodology (RSM)

The difficulty of the problem such as uncertainty conditions, ambiguity effect in various parameters, factor settings that produce the desired (maximum, minimum, or optimum) response and experimentation far from the region of optimum conditions that can be reduced by RSM which will help reduce the insignificant factors and consider the main contact factors and model the quadratic expressions based on the considerations. Central composite design (CCD) of RSM confirms the prediction capabilities in the form of second order numerical models. The 20 experimental observations of CCD with three independent input variables and second order polynomial regression quadratic model have been analyzed in the present work. The static model is fitted on the surface and based on those observations; independent variables are analyzed. The contour plots exhibit the optimum values of responses [62,63,64,65,66,67,68,69].

The COF and SWR of the GFRPA66 reinforced with 30 wt.% and 35 wt.% reinforcements are expressed by Y and the load (A), sliding speed (B), and sliding distance (C) are considered for modeling. The response can be expressed by the following equation.

β = f (A, B, C)(1)

Y = N_0_ + N_1_X_1_ + N_2_X_2_+ … +N_11_X_1_^2^ + N_22_X_2_^2^ + N_12_X_1_ X_2_ + … N_m−1,m_ N_m−1_X_m_ (2)

Initially, experiments are planned with load from 60 to 80 N, sliding velocity ranging from 0.16 to 0.22 m/s, and sliding velocity from 2500 to 3500 m. Each numerical factor is further divided into three levels: low, medium, and high. Experiments are carried out for at least three levels of each factor, with the levels being equally spaced, in order to develop a quadratic model. Table 1 displays the actual values of the factors as well as their coded levels. The optimization of process variables in RSM consists of seven distinct steps. The steps are as follows: (1) response selection (COF/SWR minimize), (2) variable selection and assignment of codes, (3) development of experimental design for minimizing the tribological properties, i.e., COF/SWR, (4) analysis of regression, (5) response development i.e., quadratic polynomial formation, (6) development of a 2D contour plot or 3D surface of the observed response surface, and finally (7) analysis of the optimum operating conditions. The experimentations on given optimal standard settings is used to validate the mathematical model generated by the RSM approach. The various statistical parameters are validated by statistical *t*-test such as R^2^ (coefficient of determination), R^2^adj (adjusted R^2^) and R^2^pred (predicted R^2^). Table 1 gives the process parameters and their levels, adopted in the present investigation.

## 4. Result and Discussion

### 4.1. Effect of Applied Load, Sliding Velocity and Sliding Distance with 30 and 35 wt.% Glass Fiber Weight on Coefficient of Friction

**Figure 3:**
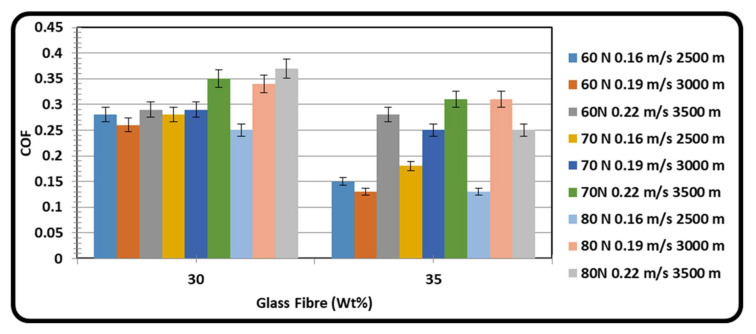
Effect of glass fiber content on COF at varying loads, sliding velocities, and sliding distances.

At different applied load conditions, sliding velocity and sliding distance, the coefficient of friction varying with respect to glass fiber content are as shown in Figure 3. The COF of GFRPA66 with 30 wt.% glass fiber decreases initially and with the further increase in the load and sliding velocity, it becomes high due to loss of transfer film on the specimen. The fiber comes out of the specimen surface by loss of transfer film, which in turn increases the friction between the exposed glass fiber and the disc. At the same time, the increased sliding velocity also increases COF due to increased temperature between the glass fiber and the disc.

In the case of GFRPA66 with 35 wt.% reinforcement, the COF decreases under all load and sliding velocity conditions attributed to the transition in material’s behavior from ductile to fragile [29,46,47]. As the glass fiber weight increases in the matrix, the glass fiber carries all of the friction load and the transfer film available on specimen surface maintains relatively lesser temperature between pin and steel disc surface. From the result it is revealed that the COF of GFRPA66 with 35 wt.% is low as compared with GFRPA66 with 30 wt.% reinforcements, since it has better transfer layer formation, increased adhesion of PA66, and low abrasion by glass fiber with less temperature between the contact surfaces. Also the elastic modulus and ultimate strength of glass fiber improve as the weight of glass fiber increases [70,71,72,73].

### 4.2. Effect of Applied Load, Sliding Velocity and Sliding Distance with Constant Glass Fiber Content 30% and 35% Weight on SWR

**Figure 4:**
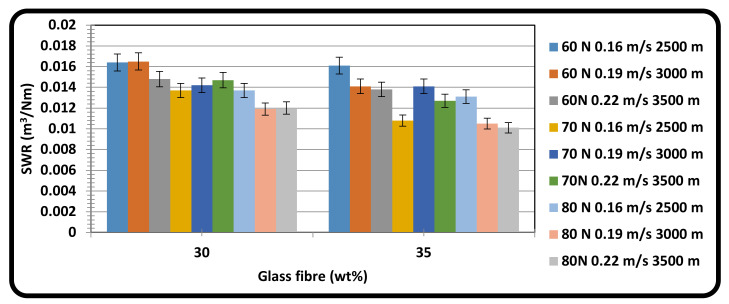
Effect of glass fiber content on SWR at varying loads, sliding velocity, and sliding distance.

Figure 4 shows the SWR variation with glass fiber content such as 30 wt.% and 35 wt.% reinforcements at different loads, sliding velocities, and sliding distances. From the graph, the SWR of GFRPA66 with 30 wt.% reinforcement range from 0.0120 m^3^/Nm to 0.0164 m^3^/Nm. Similarly, the SWR of GFRPA66 with 35 wt.% range from 0.0101 m^3^/Nm to 0.0161 m^3^/Nm. In the case of GFRPA66 with 35 wt.%, the SWR has decreased due to the increase in glass fiber content. The SWR also increased due to the increase in load, sliding velocity and sliding distance in the case of GFRPA 66 with 30 wt.% reinforcement due to stronger contact between the surfaces (such as between the pin and rotating disc) [74,75,76,77,78]. From this contact, more heat was developed between the surfaces by visco-elastic property and SWR also increased. T Trzepiecinski et al., have also iterated the significance of studying the basic concepts related to the material losses in polymer based composite materials and have reported that the heating effect is also an important factor in analyzing the material removal process, especially for the aerospace applications [79,80].

Also, when the fiber peels out by removal of transfer layer in GFRPA66 30 wt.% after an increase in the load, sliding velocity, and sliding distance, greater contact occurs between the two relatively hard surfaces (EN31 steel and peeled glass fiber) resulting in severe abrasive friction. In case of GFRPA66 35 wt.%, the fiber reinforcement does not peel out as the transfer film is retained even at increased load, sliding velocity and sliding distance, thereby resulting in contact between a hard surface (EN31 steel) and another relatively soft (polymer) material, which corresponds to a soft abrasive friction mechanism, and a lesser SWR.

### 4.3. Optimization of Experimental Condition of GFRPA66 30 wt.% and GFRPA66 35 wt.%

Using Design—Expert 13.0 software, optimization of experimental conditions were carried out to minimize the COF and SWR of the composite specimens. The process variables were wisely chosen to optimize the load, sliding velocity, and sliding distance. The optimization program was used to determine the highest level of desirability, and then different numerical combinations were searched in order to maximize the model functions. The optimized conditions of GFRPA66 with 30 wt.% reinforcements and their response for Minimum COF and SWR was obtained at a load of 80N, a sliding velocity of 0.22 m/s and a sliding distance of 3500m. The optimized conditions of GFRPA66 with 35 wt.% reinforcements and their response for minimum COF and SWR was obtained at a load of 70 N, a sliding velocity of 0.19 m/s and a sliding distance of 3000 m. The obtained optimum conditions were further validated by an additional set of experiments to confirm the minimization. This confirmatory run validated the model’s accuracy, COF and SWR datasets using the model equation. The model assumed theoretical values were well in agreement with experimentally observed response levels. This indicates the response surface models’ precision and accuracy.

#### Development of Response Surface Models of Composite Specimens

Table 2 and Table 3 shows the analysis of variance (ANOVA) results for both the models. The “F-value” of GFRPA66 composite with 30 wt.% reinforcement and GFRPA66 with 35 wt.% reinforcement for the developed model was found to be 96.25 and 44.54 for COF and 17411.57 and 48.87 for SWR, respectively, indicating that both the models are statistically significant.

Large F values can occur due to noise, with a 0.01% chance. The “Prob > F” values are less than 0.0500, indicating that both model terms are significant. Model terms for GFRPA66 30 wt.%—A, B, C, AC, A^2^, B^2^, C^2^ and GFRPA66 35 wt.%—A, B, C, AC, B^2^, C^2^ are found to be significant for COF, respectively. In the case of SWR also, model term for GFRPA66 30 wt.%—A, B, C, AB, C^2^ and GFRPA66 35 wt.%—B, C, AB, AC, BC, B^2^, respectively, are found to be significant. “p values” for all of the models were remarked to be <0.001, representing the significance level of developed models. The model terms are insignificant if the *p*-values are greater than 0.10. In addition to p value, the ability of developed models is evaluated using other statistical parameters such as R^2^ (coefficient of determination), R^2^adj (adjusted R^2^), R^2^pred (predicted R^2^), CV (coefficient of variation), etc. The R^2^ analysis for COF for GFRPA66 30 wt.% (R^2^ values-0.9886 and R^2^adj −0.9783) and COF—GFRPA66 35 wt.% (R^2^ values—0.9757 and R^2^adj −0.9538) are greater than 0.9 which ascertains its validity. In the case of SWR, R^2^ values for GFRPA66 30 wt.% (R^2^ = 0.9999 and R^2^adj. = 0.9999) and GFRPA66 35 wt.% (R^2^ = 0.9778 and R^2^adj. = 0.9578) are having a value closer to 1. Both the models’ standard deviations (SD) are found to be small (i.e., the SD for GFRPA66 30 wt.% (COF-0.0048. and SWR-0.0001) and GFRPA66 35 wt.% (COF-0.0252 and SWR-0.0069)) are very minimal. The adjusted R^2^ value is very close to the predicted R^2^ value and models are statistically accurate.

To fit the experimental outcomes attained from the design, several iterative are runs performed in connection with the arranged CCD, the response designs are subsequently modified. This resulted in a coded equation. Where, A, B, and C represent load, sliding velocity and sliding distance. Consequently, the response (i.e., minimum COF and SWR for GFRPA66 30 wt.% and GFRPA66 35 wt.% was calculated using Equations (3)–(6), respectively) [33,34,35,36,37,50].

(COF)GFRPA66 30 wt.% = +0.2866 + 0.0127 A+0.0196 B + 0.0130 C + 0.0238 AC + 0.0105 A^2^ + 0.0105 B^2^ − 0.0124 C^2^ (3)

(COF)GFRPA66 35 wt.% = +0.2646 + 0.0613 A + 0.0524 B+0.0916 C + 0.0687AC + 0.0639B^2^ − 0.0249 C^2^ (4)

(SWR)GFRPA66 30 wt.% = +0.0142 − 0.0014 A + 0.0003 B − 0.0011 C − 0.0000 AB + 0.0000 C^2^ (5)

(SWR)GFRPA6635 wt.% = +0.0186 − 0.0049B − 0.0056C + 0.0216 AB − 0.0205 AC − 0.0152BC + 0.0252 B^2^ (6)

**Figure 5:**
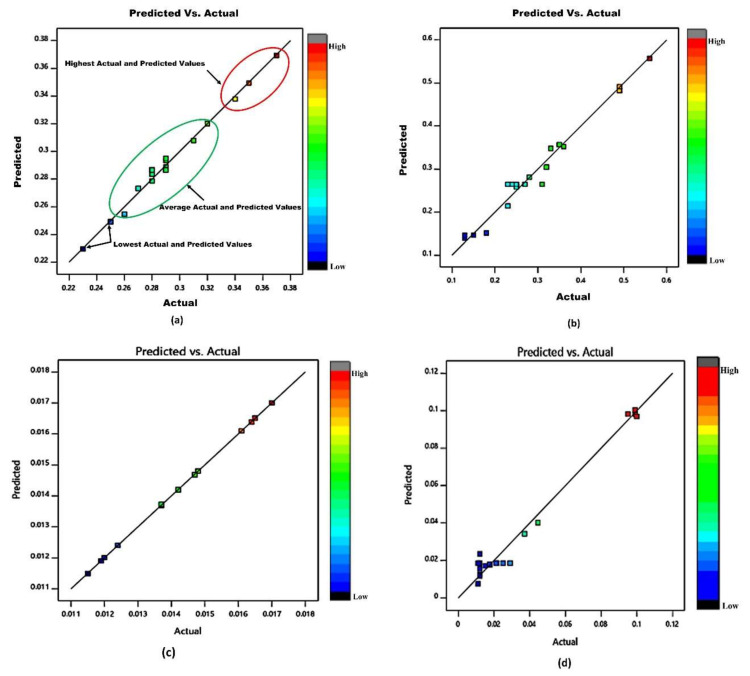
(**a**) Relationship between actual and predicted values of GFRPA66 30 wt.% COF; (**b**) relationship between actual and predicted values of GFRPA66 35 wt.% COF; (**c**) relationship between actual and predicted values of GFRPA66 30 wt.% SWR; (**d**) relationship between actual and predicted values of GFRPA66 35 wt.% SWR.

The relationship between actual values of COF for GFRPA66 30 wt.% and GFRPA66 35 wt.% and SWR for GFRPA66 30 wt.% and GFRPA66 35 wt.% and the measured responses depicted by CCD, are as shown in Figure 5a–d, and by using the estimated function values for model assessment, the differences between the actual and predicted responses are found to be close to each other.

**Figure 6:**
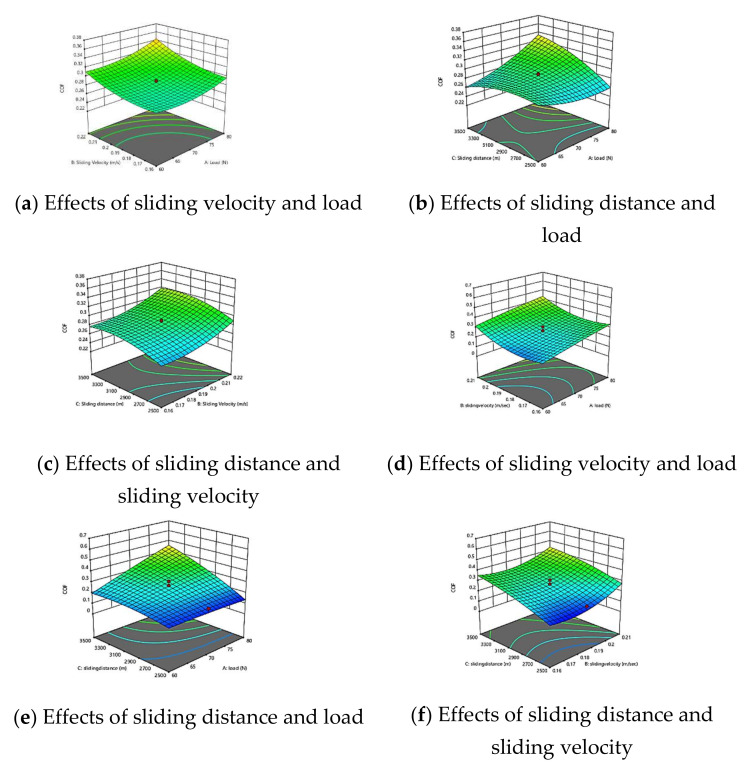
3D surface of COF model GFRPA66 30 wt.% and GFRPA66 35 wt.%; (**a**) GFRPA66 30 wt.% effects of sliding velocity and load, (**b**) GFRPA66 30 wt.% effects of sliding distance and load, (**c**) GFRPA66 30 wt.% effects of sliding distance and sliding velocity, (**d**) GFRPA66 35 wt.% effects of sliding velocity and load, (**e**) GFRPA66 35 wt.% effects of sliding distance and load, and (**f**) GFRPA66 35 wt.% effects of sliding distance and sliding velocity.

To examine the combined effect of the factors on the COF of GFRPA66 30 wt.% and GFRPA66 35 wt.%, 3-D surface plots of the regression equation were used and are presented in Figure 6a–f. The contour 3D-plot shows the plot in three different colors: the lowest value—blue; the average value—green and cyan; and the highest value—red. The highest COF was obtained due to more interaction between polyamide and the steel surface, such as frictional heating and bulk erosion. Similarly, the lowest COF was obtained due to strong control of molecular position during the run. The moderate COF was obtained due to a lower surface degradation effect.

**Figure 7:**
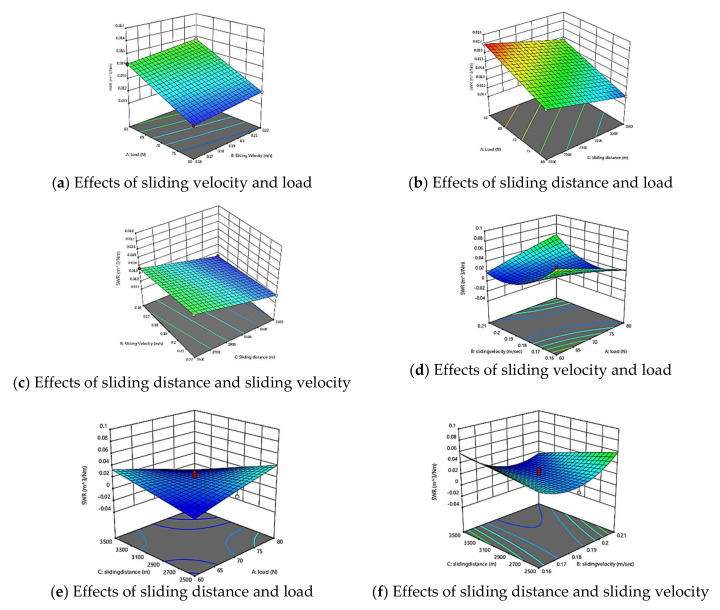
3D surface of SWR model GFRPA66 30 wt.% and GFRPA66 35 wt.%; (**a**) GFRPA66 30 wt.% effects of sliding velocity and load, (**b**) GFRPA66 30 wt.% effects of sliding distance and load, (**c**) GFRPA66 30 wt.% effects of sliding distance and sliding velocity, (**d**) GFRPA66 35 wt.% effects of sliding velocity and load, (**e**) GFRPA66 35 wt.% effects of sliding distance and load, and (**f**) GFRPA66 35 wt.% effects of sliding distance and sliding velocity.

To study the combined effect of the factors on SWR of GFRPA66 30 wt.% and GFRPA66, 3-D surface plots of the regression equation were used in Figure 7a–f. The contour 3D-plot shows the plot in three different colors: the lowest value—blue; the average value—green and cyan; and the highest value—red. The highest SWR was obtained due to the volume of the material decreased. Similarly, the lowest SWR was obtained due to strong control of molecular position during run and less frictional heating (e.g., less temperature developed between contacting surfaces). The moderate SWR was obtained due to less degradation of polyamide occurs throughout the whole material evenly.

##### Experimental Validation

In order to evaluate the numerical model, validating experiments are conducted at optimized parameters of 70 N load, sliding velocity of 0.19 m/s, sliding distance of 3000 m, and weight percentage of 35%. The results of the predictions and experiments for COF and SWR can be seen in Table 4. It is herewith reported that the experimental outcomes and predicted results are in close agreement.

### 4.4. Worn Surface Morphology for Variations in Sliding Velocity and Sliding Distance at 80N Load

**Figure 8:**
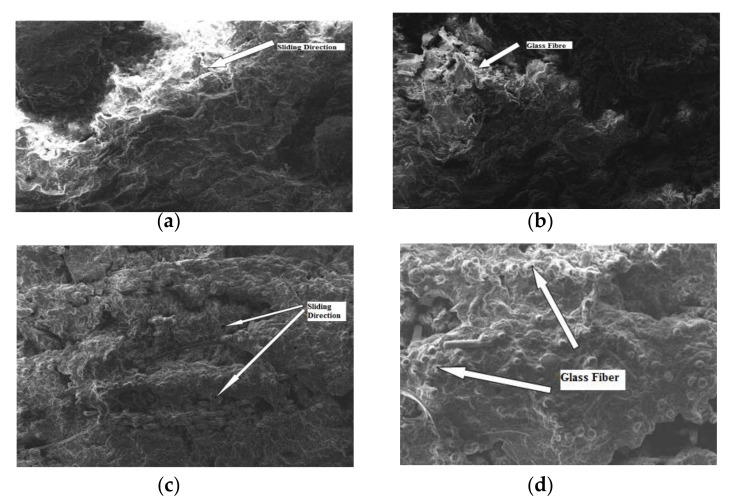
SEM image of worn surfaces of the GFRPA66 30 wt.% and GFRPA66 35 wt.%, at a load of 80 N: (**a**) GFRPA66 30 wt.%, 0.16 m/s; (**b**) GFRPA66 30 wt.%, 0.22 m/s; (**c**) GFRPA66 35 wt.%, 0.16 m/s; (**d**) GFRPA66 35 wt.%, 0.22 m/s.

Figure 8a,b shows the worn out surfaces of GFRPA66 30 wt.%, for a sliding velocity 0.16, and 0.22 m/s, sliding distance of 2500 m, and 3500 m and applied load of 80 N. It is observed that the glass fiber comes out of the surface due to surface degradation and increase the SWR. Similarly Figure 8c,d shows the worn out surface of GFRPA66 35 wt.%, for the same set of parameters. It is observed that the glass fiber does not come out of the surface and is less due to less surface erosion. Subsequently, it decreases the SWR.

## 5. Conclusions

The tribological properties of polyamide GFRPA66 30 wt.% and GFRPA66 35 wt.% composites are analyzed, and the following inferences are drawn:

- The friction coefficient and SWR decreases with increase in weight percentage of glass fiber content and lowest values are achieved for 35 wt.% of glass fiber.
- The SWR of GFRPA66 30 wt.% ranges from 0.0120 m^3^/Nm to 0.0164 m^3^/Nm for different set of process parameters. Similarly, the SWR of GFRPA6635 wt.% range from 0.0101 m^3^/Nm to 0.0161 m^3^/Nm. However, as the load, sliding velocity and sliding distance increases, the SWR decreases.
- The coefficient of friction values ranges from 0.22 to 0.37 for GFRPA66 30 wt.%, while the coefficient of friction values ranges from 0.15 to 0.31 for GFRPA 66 35 wt.% for different set of process parameters, which are carefully observed and optimized.
- The ANOVA revealed that the significant and insignificant terms used for the model (i.e., the “F-value” of GFRPA66 30 wt.% and GFRPA66 35 wt.% for the developed model) was found to be 96.25 and 44.54 for COF and 17411.57 and 48.87 for SWR, respectively, indicating that both models are statistically significant.
- The R^2^ values for COF for GFRPA66 30 wt.% (R^2^ values-0.9886 and R^2^adj -0.9783) and COF for GFRPA66 35 wt.% (R^2^ values- 0.9757 and R^2^adj -0.9538) are close to a unity. In the case of SWR, GFRPA66 30 wt.% (R^2^ = 0.9999 and R^2^adj. = 0.9999) and SWR- GFRPA66 35 wt.% (R^2^ = 0.9778 and R^2^adj. = 0.9578) are also close to unity.
- Based on the significant terms, the polynomial equations are formed and using this equation, optimized COF and SWR are estimated.
- The experimental validations signified that the predicted values are in good conformity with the actual data and the developed models are adequate.
- The SEM image showed the wear mechanism of the worn out surface of composites and glass fibers which have come out of the polyamide surface for GFRPA66 30 wt.% and is more than the GFRPA66 35 wt.% composites.

## Figures and Tables

**Table 1:** Process parameters and their levels.

Parameters	Factor Levels		
	−1	0	+1
Load (N)	60	70	80
Sliding Velocity (m/s)	0.16	0.19	0.22
Sliding Distance (m)	2500	3000	3500

**Table 2:** ANOVA table of COF for GFRPA66 30 wt.% and GFRPA66 35 wt.%.

Source	ANOVA for GFRPA66 30 wt.%					ANOVA for GFRPA66 35 wt.%				
	SS	df	MSq	F-Value	*p*-Value	SS	df	MSq	F-Value	*p*-Value
Model	0.0203	9	0.0023	96.25	<0.0001	0.2546	9	0.0283	44.54	<0.0001
A	0.0022	1	0.0022	94.51	<0.0001	0.0513	1	0.0513	80.72	<0.0001
B	0.0052	1	0.0052	223.52	<0.0001	0.0365	1	0.0365	57.54	<0.0001
C	0.0023	1	0.0023	98	<0.0001	0.0874	1	0.0874	137.68	<0.0001
AB	0.0001	1	0.0001	4.79	0.0534	0.0001	1	0.0001	0.1771	0.6828
AC	0.0045	1	0.0045	192.17	<0.0001	0.0378	1	0.0378	59.53	<0.0001
BC	0	1	0	0.5323	0.4824	0.0010	1	0.0010	1.60	0.2353
A^2^	0.0016	1	0.0016	68.28	<0.0001	0.0004	1	0.0004	0.6582	0.4361
B^2^	0.0016	1	0.0016	68.28	<0.0001	0.0567	1	0.0567	89.34	<0.0001
C^2^	0.0022	1	0.0022	94.88	<0.0001	0.0053	1	0.0053	8.37	0.0160
Res	0.0002	10	0			0.0064	10	0.0006		
Lack of Fit	0.0001	5	0	0.7611	0.6141	0.0023	5	0.0005	0.5556	0.7327
Pure Error	0.0001	5	0			0.0041	5	0.0008		
Cor Total	0.0206	19				0.2610	19			
Standard Deviation= 0.0048 R^2^ = 0.9886			Adjusted R^2^ = 0.9783 Adeq Precision = 40.7828			Standard Deviation= 0.0252 R^2^ = 0.9757			Adjusted R^2^ = 0.9538 Adeq Precision = 23.4526	

**Table 3:** ANOVA table of SWR for GFRPA66 30 wt.% and GFRPA66 35 wt.%.

Source	ANOVA for GFRPA66 30 wt.%					ANOVA for GFRPA66 35 wt.%				
	SS	df	MSq	F-Value	*p*-Value	SS	df	MSq	F-Value	*p*-Value
Model	0.0000	9	4.816 × 10^−6^	17411.57	<0.0001	0.0209	9	0.0023	48.87	<0.0001
A	0.0000	1	0.0000	92934.97	<0.0001	0.0000	1	0.0000	0.9939	0.3423
B	1.103 × 10^−6^	1	1.103 × 10^−6^	3989.13	<0.0001	0.0003	1	0.0003	6.59	0.0280
C	0.0000	1	0.0000	59745.62	<0.0001	0.0003	1	0.0003	6.84	0.0258
AB	5.000 × 10^−9^	1	5.000 × 10^−9^	18.08	0.0017	0.0037	1	0.0037	78.42	<0.0001
AC	0.0000	1	0.0000	0.0000	1.0000	0.0034	1	0.0034	70.46	<0.0001
BC	0.0000	1	0.0000	0.0000	1.0000	0.0019	1	0.0019	40.00	<0.0001
A^2^	3.624 × 10^−13^	1	3.624 × 10^−13^	0.0013	0.9718	0.0000	1	0.0000	0.5246	0.4855
B^2^	3.624 × 10^−13^	1	3.624 × 10^−13^	0.0013	0.9718	0.0089	1	0.0089	186.44	<0.0001
C^2^	4.423 × 10^−9^	1	4.423 × 10^−9^	15.99	0.0025	2.795 × 10^−6^	1	2.795 × 10^−6^	0.0587	0.8134
**Residual**	2.766 × 10^−9^	10	2.766 × 10^−10^			0.0005	10	0.0000		
Lack of Fit	2.016 × 10^−9^	5	4.032 × 10^−10^	2.69	0.1509	0.0002	5	0.0000	0.8700	0.5589
Pure Error	7.500 × 10^−10^	5	1.500 × 10^−10^			0.0003	5	0.0001		
**Cor Total**	0.0000	19				0.0214	19			
Standard Deviation = 0.00010 R^2^ = 0.9999			Adjusted R^2^ = 0.9999 Adeq Precision = 468.7422			Standard Deviation = 0.0069 R^2^ = 0.9778			Adjusted R^2^ = 0.9578 Adeq Precision = 19.0151	
MS—Sum of Square; df—Degree of Freedom; MSq—Mean Square; Res—Residual										

**Table 4:** Validation experiments of COF and SWR for PA66 GF35.

Polyamide	Values	Predicted	Experimental	Error
PA66GF Wt 35%	COF	0.3401	0.3409	0.0008
	SWR(m^3^/Nm)	0.0122	0.0128	0.0006

## Data Availability

Not applicable.
